# Can intracellular *Staphylococcus aureus* in osteomyelitis be treated using current antibiotics? A systematic review and narrative synthesis

**DOI:** 10.1038/s41413-022-00227-8

**Published:** 2022-08-12

**Authors:** Anja R. Zelmer, Renjy Nelson, Katharina Richter, Gerald J. Atkins

**Affiliations:** 1 Centre for Orthopaedic and Trauma Research, Faculty of Health and Medical Sciences, University of Adelaide, Adelaide, SA 5000 Australia; 2 Department of Infectious Diseases, Central Adelaide Local Health Network, Adelaide, SA 5000 Australia; 3 Royal Adelaide Hospital, Adelaide, SA 5000 Australia; 4 Richter Lab, Department of Surgery, Basil Hetzel Institute for Translational Health Research, University of Adelaide, Adelaide, SA 5011 Australia

**Keywords:** Diseases, Pathogenesis

## Abstract

Approximately 40% of treatments of chronic and recurrent osteomyelitis fail in part due to bacterial persistence. *Staphylococcus aureus*, the predominant pathogen in human osteomyelitis, is known to persist by phenotypic adaptation as small-colony variants (SCVs) and by formation of intracellular reservoirs, including those in major bone cell types, reducing susceptibility to antibiotics. Intracellular infections with *S. aureus* are difficult to treat; however, there are no evidence-based clinical guidelines addressing these infections in osteomyelitis. We conducted a systematic review of the literature to determine the demonstrated efficacy of all antibiotics against intracellular *S. aureus* relevant to osteomyelitis, including protein biosynthesis inhibitors (lincosamides, streptogramins, macrolides, oxazolidines, tetracyclines, fusidic acid, and aminoglycosides), enzyme inhibitors (fluoroquinolones and ansamycines), and cell wall inhibitors (beta-lactam inhibitors, glycopeptides, fosfomycin, and lipopeptides). The PubMed and Embase databases were screened for articles related to intracellular *S. aureus* infections that compared the effectiveness of multiple antibiotics or a single antibiotic together with another treatment, which resulted in 34 full-text articles fitting the inclusion criteria. The combined findings of these studies were largely inconclusive, most likely due to the plethora of methodologies utilized. Therefore, the reported findings in the context of the models employed and possible solutions for improved understanding are explored here. While rifampicin, oritavancin, linezolid, moxifloxacin and oxacillin were identified as the most effective potential intracellular treatments, the scientific evidence for these is still relatively weak. We advocate for more standardized research on determining the intracellular effectiveness of antibiotics in *S. aureus* osteomyelitis to improve treatments and patient outcomes.

## Introduction

Osteomyelitis, inflammation of the bone and bone marrow mainly caused by a microbial pathogen, is an increasing health problem. In adults, the major types of osteomyelitis are that associated with peripheral vascular disease common in diabetes mellitus, termed diabetic foot infection (DFI); that arising from an open fracture, termed fracture-related infection (FRI); and that arising from insertion of orthopedic implants, termed periprosthetic joint infections (PJI).^1,2^ The prevalence of osteomyelitis, as indicated by the documented increase in the incidence of PJI, is rising as a result of societal aging, increased demand for joint replacement surgery and the increased incidence of metabolic and perivascular disease. As an example, in Australia, there was an increase in the incidence of replacements of 1.9% in hips, 1.3% in knees and 4.9% in shoulders in 2019 compared to those in 2018. Compared to the numbers in 2003, the increase in the incidence of replacements for hips was 89.3% and for knees 133.1%. For shoulder replacements, the increase in incidence since 2008 was 189.6%.^3^ The annual joint replacement failure rate is declining, primarily due to improved wear rates of prosthesis materials; however, the infection rate is slowly but steadily increasing.^4^ Alarmingly, since the 1970s, there have been no improvements in surgical techniques to reduce the number of infections, resulting in a constant, slowly increasing infection rate of 5%–33% in hips and knees after open fractures and 1%–4% in joint replacements.^5,6^ Despite the use of optimal medical care, osteomyelitis is difficult to cure, and treatment failure rates are between 10%–40%.^7–9^

Osteomyelitis can affect one or more tissues and locations in the bone, including the overlying soft tissues, periosteum, bone marrow, and cancellous and cortical bone.^2^ Acute osteomyelitis is characterized by suppurative inflammation, with an influx of leukocytes, including macrophages and neutrophils, the latter being the most diagnostic.^2,10^ Osteomyelitic bone tissue shows evidence of inflammatory remodeling and bone destruction, most often attributed to increased osteoclast activity, as well as osteoblast and bone necrosis, particularly in the avascular sequestra.^2,11^ In a recent study of cancellous bone taken from PJI patients with a wide range of organisms, extensive bone matrix collagenolysis was observed, which could be attributed at least in part to pathogen-induced expression of matrix metallopeptidases (MMPs) by osteocytes, suggesting an additional host-derived pathologic process.^12^

Up to 75% of infectious osteomyelitis cases are caused by staphylococci, with *Staphylococcus aureus* and the coagulase-negative species *Staphylococcus epidermidis* (*S. epidermidis*) being the most common pathogens. *S. aureus* infection has the highest treatment failure rate in PJI, up to 4 times higher than that for *S. epidermidis*, and causes up to 50% of cases of osteomyelitis in PJI as methicillin-resistant *S. aureus* (MRSA).^13–15^

*S. aureus* has the ability to form small-colony variants (SCVs), which are a reversible phenotypic variation manifesting as a quasidormant state. Compared to the corresponding wild-type (WT) strain, they are characterized by a smaller colony size, slow and linear growth, altered metabolism and the expression of a reduced number of virulence factors.^16^ These alterations lead to lower susceptibility to antibiotics and impaired eradication by the immune system.^17^ SCVs grow mostly in protected environments, such as in biofilms and in intracellular reservoirs;^18,19^ their development can be induced by antibiotic treatments, including trimethoprim-sulfamethoxazole (TMP-SMX) and aminoglycosides;^20,21^ and they are frequently found in chronic infections.^22,23^ The intracellular persistence of SCVs is more successful than that of the corresponding isogenic WT strain in nonprofessional phagocytes.^20,24^ In a clinical study, SCVs were found in 34% of PJI patients, with a prevalence of 55.3% of all isolated bacteria, indicating the higher diversity of SCVs than the WT strain.^14^ It was reported in a recent study that *S. aureus* infection of osteocytes was a feature of clinical PJI and that in an experimental model of intracellular infection, SCVs rapidly appeared and became the dominant detectable phenotype within a 6-day infection period.^25^ Due to the important role of SCVs in intracellular and persistent osteomyelitis infections, appropriate treatment regimens should be selected that target pathogens, including SCVs, without inducing their development.

Of the bone-resident cell types, intracellular infections have been demonstrated in osteoblasts, osteoclasts and osteocytes in vitro and in vivo,^25–34^ and the ability of intracellular *S. aureus* to cause osteomyelitis has been demonstrated in vivo.^35^ There are recent reviews describing the evasion of host defenses by *S. aureus,*^36^ the role of *S. aureus* in osteomyelitis^37^ and the intracellular persistence of *S. aureus* in osteomyelitis.^38^ This finding demonstrates the role of intracellular persistence, especially of *S. aureus*, in osteomyelitis and supports the call for developing and evaluating effective treatment strategies.

Remarkably, two-thirds of available antibiotics are reported to be ineffective against intracellular infections^39^ for various reasons, including the following:

- low drug concentration in infected tissue due to limited drug penetration;
- subtherapeutic intracellular concentration of antibiotic due to limited drug uptake and/or high clearance rate;
- lack of drug-pathogen interactions due to spatial distance between antibiotic and bacteria in the same intracellular compartment;
- low intracellular activity of antibiotic;
- low bacterial susceptibility to antibiotics.

To better predict treatment efficacy, data from multiple sources have been combined, for example, pharmacokinetic data to determine the concentration of the antibiotic in bone,^40,41^ the uptake and clearance of drugs inside specific cells^42^ and the effect of drugs on host cell metabolism or antibiotic activity at various pH levels.^43^ However, it is still uncertain whether specific treatments actually reduce intracellular bacterial numbers. Osteomyelitis is best modeled in infected cells using antibiotic concentrations that are known to be achieved in the bone.

A recent comprehensive review highlighted the effects of antibiotics on human neutrophils and the intracellular effectiveness of antibiotics.^44^ Nevertheless, this study did not focus on osteomyelitis and therefore did not consider the local antibiotic concentration in the bone or tissue other than blood, nor, to our knowledge, does any other review.

This review summarizes and evaluates the current literature regarding the effectiveness of antibiotic treatments against intracellular *S. aureus* infection of osteomyelitis-relevant cells and in vivo studies to build the basis for formulating treatment guidelines for osteomyelitis. Furthermore, we identify differences in the methodologies utilized and advocate for standardized in vitro models, as well as highlight promising treatment options that should be investigated further.

## Methods

Systematic searches in two databases were performed to find literature about the treatment of osteomyelitis with intracellular *S. aureus* infection with antibiotics. The search terms for PubMed were intracellular + *S. aureus* + antibiotic + bone or osteoblast or osteomyelitis, intraosteoblastic + staphylococci, and osteoblast + staphylococcus + intracellular. The search terms for Embase were intracellular AND (‘antibiotic’/exp OR antibiotic) AND (‘bone’/exp OR bone OR ‘osteoblast’/exp OR osteoblast OR ‘osteomyelitis’/exp OR osteomyelitis) AND (‘staphylococcus aureus’/exp OR ‘staphylococcus aureus’). Ultimately, 131 abstracts from PubMed and 118 abstracts from Embase were screened for inclusion.

**Fig. 1:**
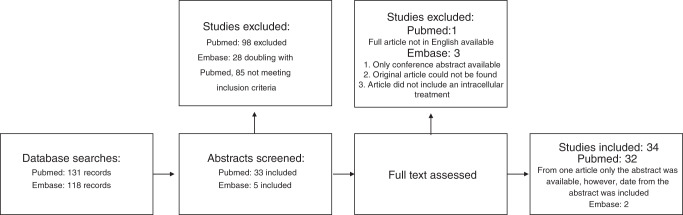
Schematic representation of the literature screen performed. Four articles are not included in the tables, since they did not measure the reduction of the number of colony-forming units (CFU) by the antibiotic as an outcome

Articles that examined *S. aureus* as the pathogen for an in vitro or in vivo intracellular infection in the context of osteomyelitis that were treated with classic antibiotics or antibiotic formulations together with other treatments if there was an antibiotic comparison were included. Articles reporting on other pathogens or solely on nonantibiotic treatments were excluded. This approach identified 38 articles for full-text assessment. Four of these were excluded, 3 because of unavailability of the original articles (one conference abstract, one was a non-English language article, and one could not be found on the journal website) and one because the study did not use an intracellular treatment, resulting in 34 articles included in this review. Figure 1 shows an overview of the included literature.

## Results

This review is divided into subsections according to the three major antibiotic mechanisms of action: protein biosynthesis inhibition, bacterial enzyme inhibition and cell wall disruption. Information is provided on antibiotic profiles, usage in osteomyelitis, antimicrobial resistance, bone concentration of antibiotic, intracellular penetration, extracellular minimum inhibitory concentration (MIC) and intracellular effectiveness. For some antibiotics, enhanced drug delivery systems have been tested in intracellular models, which are also discussed. Only a few studies with preclinical in vivo data could be included in this review at the end of each section, before a concluding statement about the antibiotic group. To our knowledge, there are no published clinical data showing the intracellular activity of antibiotics in human osteomyelitis patients. To compare different studies, all concentrations were adapted to units of micrograms per milliliter (μg·mL^−1^) or μg·g^−1^ if no volume percentage could be found, and the intracellular effectiveness was measured by logarithmic colony-forming unit (CFU) reduction.

### Protein biosynthesis inhibitors

#### Lincosamide: clindamycin

Clindamycin is commonly used for the treatment of osteomyelitis due to its activity spectrum against gram-positive cocci (especially *Staphylococcus*, including erythromycin-resistant *Staphylococcus*, and *Streptococcus*), gram-negative cocci and intracellular bacteria (*Chlamydia* and *Rickettsia* species) and its good bone penetration properties.^45,46^ With some exceptions (i.e., *Bordetella pertussis, Campylobacter, Chlamydia, Helicobacter*, and *Legionella* species), clindamycin is not effective against gram-negative bacilli.^46–48^

The antibacterial effect of lincosamides is achieved by the inhibition of elongation during protein synthesis by interfering with peptide bond formation between the A- and P-sites of the tRNA of the 50 S subunit of bacterial ribosomes.^48,49^ This common binding site shared by multiple drugs can lead to cross-resistances, most frequently macrolide, lincosamide and streptogramin B (MLS_b_) cross resistance (see 4.1.4 MLS_b_ resistance).

In osteomyelitis patients, the bone penetration of clindamycin as a monotherapy is rapid and high, with a bone:plasma ratio between 0.2-0.6 leading to bone concentrations between 1.4-9.6 μg·mL^−1^. However, cotreatment with rifampicin can reduce the clindamycin bone concentration to a subtherapeutic level.^40,41,50–52^

In vitro, clindamycin treatments against *S. aureus* showed a high variation in intracellular activity between <1-log to 5-log reduction if cells were treated immediately after the infection.^43,53–58^ No measurable reductions were reported with delayed treatment after 12 h^54^ or 7 days.^55^ Clindamycin showed limited effects against *S. aureus* SCVs, reducing the number of intracellular bacteria by 1-log after 72 h and extracellular bacteria by 2-log after 24 h in THP-1 macrophages.^17^ Furthermore, clindamycin was also reported to induce SCV formation.^55^ The concentration of clindamycin used in the studies varied between 1.3–20 μg·mL^−1^, but most commonly 4 μg·mL^−1^ was used,^17,43,53–58^ which is a concentration that might realistically be reached in the bone. The MICs for various *S. aureus* strains were between 0.004–8 μg·mL^−1^ at pH 7. However, acidic pH can increase the MIC fourfold or more to >38 μg·mL^−1^ compared to 4–8 μg·mL^−1^ in the same strain at pH 7.^43^ This indicates that the treatment efficacy in patients depends on the pH of the compartment where the antibiotic and bacteria interact. Therefore, increasing the intracellular pH could be a strategy to improve treatment efficacy. As an example, combining clindamycin with hydroxychloroquine in vitro resulted in an intracellular CFU reduction of 2.7-fold.^43^ Moreover, clindamycin incorporated in a calcium phosphate powder showed increased antibacterial activity compared to that of the standard formulation after 24 h and 48 h, suggesting that smart drug delivery systems have the potential to improve treatment effectiveness against intracellular bacteria in osteomyelitis.^57^

From the current in vitro data, no conclusion can be made regarding the effectiveness of clindamycin in treating intracellular *S. aureus* in osteomyelitis. Most studies showed limited effectiveness that relied on immediate treatment after the infection, which is typically not possible in a clinical setting. Therefore, clindamycin is likely to be ineffective against intracellular *S. aureus* infections in osteomyelitis.

#### Streptogramins: quinupristin/dalfopristin

Quinupristin/dalfopristin is a 30:70 combination of two Streptomyces-derived streptogramin antibiotics (streptogramin A and streptogramin B), which have a synergistic bactericidal effect. The combination is used against staphylococci, streptococci and *Enterococcus faecium*^59,60^ and inhibits protein biosynthesis at the 50 S subunit of bacterial ribosomes. Dalfopristin (streptogramin A) binds to the A- and P-sites of the peptidyl transfer center of the ribosome and changes the ribosomal conformation, enhancing the binding of quinupristin (streptogramin B) approximately 100-fold.^59,61^ Furthermore, dalfopristin hinders peptidyl transfer elongation by inhibiting tRNA binding and translation. Similar to other MLS_B_ antibiotics, quinupristin obstructs the elongation and particularly the translocation and extension of proteins and causes the release of incomplete protein chains.^61–63^

Currently, quinupristin/dalfopristin (market name Synercid) has been approved only for skin and soft tissue infections,^64^ but it has been used off-label in osteomyelitis patients. A case report described the successful treatment of a patient with a vancomycin-resistant enterococcus (VRE) infection,^65^ and a phase I clinical trial concluded with the successful treatment of 32 out of 40 patients, defined as clinical cure or improvement.^66^

Another drug combination is pristinamycin, composed of pristinamycin IA (streptogramin B) and pristinamycin IIA (streptogramin A). It is effective against MRSA, erythromycin-resistant staphylococci and streptococci and has been used effectively against osteoarticular infections and osteomyelitis.^67,68^

In our search, only one in vitro study on the effect of quinupristin/dalfopristin against intracellular *S. aureus* SCVs was found, which reported an intermediate effectiveness of a 2-log reduction against intracellular SCVs compared to a 3-log reduction against extracellular SCVs. Remarkably, a sudden onset of the antibacterial effect after 72 h intracellularly and after 5 h extracellularly was noted, which did not increase further at later time points. Even though the MICs against all bacterial phenotypes were the same (0.5 μg·mL^−1^), the antibiotics were more effective against the WT and the revertant strains than against the SCV form in an experimental infection in THP-1 macrophages.^17^ Although quinupristin/dalfopristin is not commonly used to treat osteomyelitis and there are only limited data about its intracellular effectiveness, it seems to be a promising treatment option; however, further studies are needed.

#### Macrolides: azithromycin, telithromycin, erythromycin, spiramycin

Macrolides are antibiotics produced by gram-positive Actinomycetes and were discovered in the 1950s.^69^ A large number of macrolide antibiotics are now available for standard medical care and specifically as an alternative therapy for penicillin-allergic patients.^70^

Similar to penicillins, macrolides are mainly effective against gram-positive bacteria (e.g., staphylococci and streptococci) but are also effective against some gram-negative species (*Neisseria gonorrhoeae*, *Haemophilus influenzae*, *Bordetella pertussis* and *Neisseria meningitis*) and mycoplasma species.^71,72^

Macrolides inhibit bacterial protein synthesis by hindering tRNA attachment to the peptidyl transferase of the 50 S subunit of bacterial ribosomes, thus disrupting polypeptide chain elongation. The shared target with linezolid and streptogramins B can lead to MLS_B_ resistance.^49,72,73^

Macrolides penetrate well into macrophages, with an intra- to extracellular ratio between 8.6–50.^74,75^ The bone concentration has a high variation, with bone:serum ratios between <0.05 and >3 and a median of 0.91.^40^ Azithromycin and telithromycin penetrate the bone best, with bone:plasma ratios of 2.5–6.3 and 1.5–2.6, while erythromycin and spiramycin show low bone penetration, with bone:plasma ratios of 0.18–0.28 and 0.047.^40^ The MICs of macrolides are pH sensitive, with up to a > 100-fold increase at pH 5 (0.5 μg·mL^−1^ for azithromycin, 0.06 μg·mL^−1^ for telithromycin) compared to those at pH 7 (512 μg·mL^−1^ for azithromycin, 4 μg·mL^−1^ for telithromycin), and their minimum bactericidal concentrations (MBCs) are much greater than their MICs, up to >30-fold higher (MBC: 2 μg·mL^−1^ compared to MIC: 0.06 μg·mL^−1^),^74–76^ leading to a high risk of inducing the development of persister phenotypes. Against intracellular *S. aureus* in macrophages, azithromycin and telithromycin showed less than a 1-log reduction to no measurable effect and only at very high dosages, although both antibiotics exhibited a dose-dependent extracellular effect.^74,75^ In osteoblasts, erythromycin showed some effectiveness when administered immediately but not after a 12 h delay.^54^ Since different antibiotics have been used in experiments with macrophages than those with osteoblasts and with the low number of studies reported, the difference in susceptibility of intracellular *S. aureus* to macrolides may be related to the specific drug tested rather than the cell type.

Even though macrolides seem to penetrate the bone and bone cells well, the little available evidence regarding intracellular *S. aureus* makes it difficult to discern if they are an effective treatment against intracellular *S. aureus* in an osteomyelitis setting.

#### MLS_B_ resistance

MLS_B_ resistance defines a cross-resistance between macrolides, lincosamides and streptogramin B due to a similar mode of action, targeting the bacterial 50 S subunit of the ribosomes, more specifically the 23 S ribosomal RNA segment at the peptidyl transferase center.^77^

Streptococci, enterococci and staphylococci are the main carriers of MLS_B_ resistance determinants, but they can also be expressed by other gram-positive and gram-negative species.^47^ Resistance can be constitutive (cMLSB) (especially in staphylococci) or induced (iMLSB).^78^ There are three main mechanisms of resistance: (1) methylation (esp. streptococci and enterococci) or mutation (esp. *E. coli*) that prevents target binding to most MLS_B_ antibiotics,^47,79^ (2) expression of efflux pumps to remove the antibiotic from the cell, which can be drug-specific,^80,81^ and 3) drug inactivation by esterases and phosphotransferases, which is a drug-specific mechanism that does not always lead to cross-resistance.^82^

#### Oxazolidinones: linezolid, tedizolid, radezolid

Oxazolidinones are a group of synthetic drugs with excellent oral bioavailability, making them easy for patients to use.

These bind to the 50 S subunit of the bacterial ribosome and inhibit the initiation phase of protein biosynthesis by preventing the formation of the initiation complex.^83^ Even though the binding site is closely related to MLS_B_ cross-resistant antibiotics, to date, no cross-resistance has been observed.^84,85^ Resistances determinants are rare but include a point mutation (*G2576T*) in staphylococci and *E. faecium*^83^ and G244 methylation, which reduces antibiotic binding, as well as an ABC transporter in *Streptococcus pneumoniae.*^86^

Oxazolidinones are mostly used against gram-positive bacteria (esp. streptococci, enterococci, and *S. aureus*), particularly for vancomycin-resistant strains and MRSA, including in osteomyelitis.^45,87–93^

Linezolid penetrates well into the bone, with a bone:plasma ratio of 0.2–0.6, leading to bone concentrations of 4–9 μg·mL^−1^.^40,41,53,94^ The intracellular uptake varies among the compounds, with an intra- to extracellular ratio of 0.5 in THP-1 macrophages for linezolid and between 9.3–14.4 in other cell types (9.8 for osteoblasts) for radezolid.^43,74,95^

Radezolid is a newer compound that passed phase 2 clinical trials in 2008 and 2009 but has not been used to date for treating osteomyelitis. However, it has already been tested for its intracellular activity against *S. aureus* in one study in multiple cell types (including osteoblasts), compared to that of linezolid.^95,96^ The superiority of radezolid showed an up to 8-fold reduced MIC against *S. aureus* (0.25–2 μg·mL^−1^ for radezolid compared to 1–16 μg·mL^−1^ for linezolid), as well as an approximately 10-fold higher intracellular accumulation. Similar to linezolid, it shows a reduced intra- to extracellular activity ratio, which compared to that of linezolid is higher when measured as drug concentration but is comparable when dosages similar to the MIC are compared, with an overall bacterial reduction of approximately 1-log.^95^

The effectiveness of oxazolidinones seems to be dependent on the specific formulation, the host cell type examined, and the bacterial strain being targeted. For linezolid, two studies with THP-1 macrophages reported a maximal intracellular reduction of *S. aureus* of 1-log and only slightly greater effectiveness against extracellular bacteria, independent of the growth phenotype.^17,74,95,97^ In contrast, linezolid in osteoblasts caused an up to 3-log reduction in intracellular *S. aureus* levels, even when the treatment was delayed until 7 days after the infection. However, this effect was observed only for certain strains, and some results were statistically significant but showed only 1-2-log reductions.^43,53,55,95,98,99^ No difference in the induction of or effectiveness against SCVs has been reported for linezolid.^17,53,55^ The MICs of the observed strains were between 1–4 μg·mL^−1^, with higher values in an acidic environment and for the WT compared to a those with pH of 7 and against SCVs.^17,31,43,53,74,95^ MBCs measured in one study showed a 16-fold higher MBC than MIC (MBC: 32 μg·mL^−1^ compared to MIC: 2–4 μg·mL^−1^).^74^ Tedizolid was shown to reduce the intracellular bacterial load for two-thirds of *S. aureus* strains tested by 1-log, which was similar to linezolid.^98^ Overall, the above MICs were close to the expected concentrations in the bone, and the treatments used were between 2–20 μg·mL^−1^, which may explain the high variability of the responses reported for different strains.

The use of a lipid-polymer hybrid nanoparticle loaded with linezolid improved the activity of linezolid by 35%–65% against biofilms and increased intraosteoblastic CFU reduction up to 2-log at concentrations expected in the bone.^99^ Linezolid-loaded exosomes increased extracellular and intracellular bacterial clearance in an in vitro macrophage model even further, leading to a 2-log higher intracellular clearance compared to that with the traditional formulation.^97^

In vivo, linezolid treatment of *S. aureus* intracellular osteomyelitis in rats was successful in 85%–90% of cases.^31^ Modification of the drug delivery system could increase the treatment success further, as some studies indicate. The linezolid level in the bones of rats was improved 4-fold by using lipid-polymer hybrid nanoparticles loaded with linezolid,^99^ and linezolid-loaded exosomes increased intracellular bacterial clearance in a mouse model by 2-log compared to that with the traditional formulation.^97^

Linezolid is one of the best studied antibiotics against intracellular *S. aureus*, particularly in osteoblasts. Linezolid seems to be effective in osteoblasts in vitro as well as in preclinical in vivo studies, and its effectiveness can be enhanced by embedding the antibiotic in a smart drug delivery system. Therefore, linezolid should be considered as a treatment option in osteomyelitis with a suspected intracellular *S. aureus* infection.

#### Tetracyclines/glycylcycline: doxycycline, tigecycline

Tetracyclines, such as doxycycline, are derived from *Streptomyces* actinobacteria and are currently mostly used in semisynthetic form.^100^ They have remarkable broad antibacterial activity, including against gram-positive, gram-negative and cell wall-deficient bacteria, as well as mycobacteria, protozoa and parasites.^101^

However, tetracyclines are active only against proliferating bacteria. The main mechanism of action is by binding to the 30 S ribosomal subunit, which prevents tRNA binding to the A-site, resulting in the disruption of protein translation. Other mechanisms may include (to a lesser extent) binding the 50 S ribosomal subunit and *16* *S* and *23* *S* rRNA.^102^

The emergence of multiple resistance strategies by bacteria has led to a decline in the usage of tetracyclines. These resistance mechanisms include (1) most commonly, efflux pumps, especially for first-generation tetracyclines, (2) ribosomal protection proteins (RPPs), which can block binding to tRNA even though tetracycline is already bound to the ribosome or cause detachment of the drug from its binding site, (3) enzymatic inactivation with NADPH-dependent oxidoreductase, and (4) reduced cell wall permeability.^103,104^

To overcome such resistance mechanisms, glycylcyclines were developed from tetracyclines; these have the same mechanism of action but are less susceptible to efflux pumps and RPPs and are now used against multidrug-resistant (MDR) strains, such as MRSA, VRE, and extended-spectrum beta-lactamase (ESBL)-resistant bacteria. To date, the only glycylcycline drug on the market is tigecycline.^105^

Doxycycline is regularly used to treat osteomyelitis, and tigecycline has been successfully used in some cases.^45,106,107^

Tigecycline has a bone:plasma ratio between 0.35–1.8 (measurements with newer methods suggest rather higher values), leading to a bone concentration of at least 0.3 μg·mL^−1^.^40,53^ Doxycycline has a lower bone:plasma ratio of 0.02–0.85 and reaches concentrations of 0.13–2.6 μg·mL^−1^ in the bone.^41,107^ Good bone penetration is thought to result from the calcium-binding ability of tetracyclines. Doxycycline and tigecycline have similar MICs at pH 7 (0.012 5 and 0.062 5–1 μg·mL^−1^, respectively), but the MIC of tigecycline seems to be more pH dependent (4-fold difference at pH 5) than that of doxycycline.^17,43,53^

The effectiveness of tigecycline appears to be independent of bacterial phenotype, intra- or extracellular location^17^ and the dosage of the drug.^53^ However, effectiveness was dependent on the exposure time, the bacterial strain^108^ and the cell type infected, with tigecycline being more effective against an intracellular infection of osteoblasts (up to 4-log killing) than of that of macrophages (1-log reduction).^17,53,108^

Even though the low MIC, high bone penetration rate and relatively high intracellular effectiveness against *S. aureus* seem to be promising, the lack of comprehensive data precludes a clear evaluation of the possible usage of doxycycline and tigecycline against intracellular *S. aureus* infections in osteomyelitis.

#### Fusidic acid

Fusidic acid is a steroid antibiotic extracted from the fungus *Fusidium coccineum*^109^ and is mainly used against staphylococci, other gram-positive bacteria and some gram-negative strains, such as *Neisseria* spp., *Bordetella pertussis*, and *Moraxella catarrhalis.*^110^ It is also a protein biosynthesis inhibitor and binds to the elongation factor EF-G on ribosomes.^111^

Due to the rising incidence of fusidic acid resistance, it is often used systematically in combination with other antibiotics but should not be combined with fluoroquinolones or rifampicin due to antagonistic effects.^112–114^ Rifampicin reduced the concentration and effectiveness of fusidic acid by 40%–45% in patients,^112^ likely due to induction of Cyp3A4 and pGP activity by rifampicin. However, this combination is still very commonly used.

Known resistance determinants are target mutations that reduce binding affinity and the production of proteins that detach fusidic acid from its binding site.^115^ In osteomyelitis, fusidic acid is used off-label due to the low prevalence of resistance.^116^ In Asia, 80 cases of bone and joint infections have been treated with fusidic acid, with a 90% success rate, between 2000 and 2012.^117^ An Australian study reported a success rate of 88% for one year in 20 osteomyelitis patients.^118^

Fusidic acid penetrates the bone well, with bone:plasma ratios of up to 0.94, and reaches bone concentrations of 15–45 μg·g^−1^,^40,107^ which are much higher than its MIC of 0.03–0.125 μg·mL^−1^.^17^ There is only one intracellular study of fusidic acid in macrophages, showing that the MIC and effectiveness strongly depend on the bacterial phenotype. When applied against intracellular SCVs, the CFU reduction was less than 1-log, compared to more than 1-log against the WT and revertant forms. A maximum of 1-log CFU reduction against extracellular SCVs can be achieved.^17^

With such little data about the intracellular effectiveness of fusidic acid, no conclusions can be drawn for the treatment of osteomyelitis and further studies are needed.

#### Aminoglycosides: gentamicin

Aminoglycosides are derived from *Streptomyces griseus*, and while most are active against gram-negative bacteria, they have often been used in in vitro models against intracellular *S. aureus.*^119,120^ Aminoglycosides disrupt protein synthesis by binding to 30 S ribosomal subunits, interfering with control mechanisms between the tRNA and ribosomes, leading to incorrect amino acid insertion and the production of nonfunctional proteins, as well as bacterial cell membrane disruption.^121^ Bacterial resistance mechanisms include (1) enzymatic modification or inactivation of the aminoglycosides, (2) efflux pump-based drug removal, (3) decreased membrane permeability, and (4) modification of the drug target.^122^

In osteomyelitis, gentamicin is mainly used in beads to prevent and treat infection; however, this treatment is controversial due to the possible induction of the development of resistant and/or persistent bacteria.^123^

Aminoglycosides do not penetrate well into host cells, which is why they are often used to clear extracellular bacteria, such as in the in vitro gentamicin protection assay.^124^ Even though a low cell:plasma ratio of 4.4–6.8 has been measured in macrophages, this is comparable to other antibiotics with demonstrable intracellular effects, such as oxacillin and linezolid.^74,75^ Furthermore, the penetration into the bone is very low, with barely detectable levels being described.^41^ Overall, gentamicin showed a much lower effectiveness intracellularly than extracellularly.^17,74,75,125^ In contrast, only one study found a small difference between the intra- and extracellular effectiveness.^108^ In most studies, less than a maximal plasma concentration of 20 μg·mL^−1^ was used;^55,74,75,108^ however, one study investigated the effect of 200 μg·mL^−1^,^126^ which is much higher than the concentration that can be reached in the bone. The antibiotic effect was both time-,^17^ and dose-dependent^75,126^ and had an effect when given immediately similar to that of a treatment started after 7 days.^55^ It is well known that aminoglycosides can induce SCV formation^29,55,127,128^ and that they are more effective against WT than SCV phenotypes, specifically if the SCV formation is aminoglycoside induced, which is mostly reflected in much higher MICs of SCVs than of the WT form (e.g. 32/64 μg·mL^−1^ compared to 1 μg·mL^−1^).^17,125^

The MIC of gentamicin against *S. aureus* is strongly pH dependent, with an up to 64-fold increase at pH 5 compared to that at pH 7.^75,125^ How SCV formation is induced, either by aminoglycosides or other antibiotics or factors, can influence the MIC, with up to 128-fold increased activity compared to that against the WT form.^17,74,125^

Advanced drug delivery systems, such as nanoparticles, showed an improved effectiveness in mouse osteoblasts and macrophages.^129^

In a mouse model, gentamicin could not significantly reduce the CFU count in an acute or chronic infection state,^55^ but the antibiotic effect could be increased by a nanoparticle system.^129^

In summary, gentamicin is one of the best studied antibiotics for its intracellular activity against *S. aureus*. Even though it seems to have some effect against intracellular bacteria in vitro and in vivo, the low penetration into the bone, as well as the risk of inducing resistance and persistence must be considered when used clinically. Therefore, treatment with gentamicin should always be performed with caution and only when bacterial adaptation is monitored.

A summary of the data relating to antibiotics with protein biosynthesis inhibition as a mode of action is shown in Table 1.

**Table 1:** Overview of protein biosynthesis inhibitors tested against intracellular *S. aureus*

Antibiotic Class	Antibiotic	Bone: plasma ratio	Bone concentration	Intra: extracellular ratio	MIC at ca pH5 (μg·mL^−1^ or mg·L^−1^)	MIC at ca pH7 (μg·mL^−1^ or mg·L^−1^)	Intracellular effectivity against *S. aureus*		Model tested	
								Bone cell	Other cells	In vivo
Lincosamides	Clindamycin	0.40–0.67^107^ 0.21–0.44^40,191^ 0.4^51^ 0.35^53^	2.6–5 μg·g^−1^ ^107^ 2.6 g·g^−1^ ^51^ 1.4 μg·mL^−1^ ^50^ 9.6 μg·mL^−1^ ^41^ 4 μg·mL^−1^ ^53^		≥32^43^	0.125^17^ 0.032^53^ 0.004^54^ 4–8^43^	5-log^53^ Only effective immediately, not after 12 h^54^ 1-log immediately, not after 7 days^55^ <1-log^43,56–58^ 1-log SCV^17^	MG63^43,53^ NHOst^54^ Normal Mouse OBs^54^ pHOBs^55^ MC3T3^57^ UMR106^58^	THP-1^17^ Primary polymorphonuclear leukocytes^56^	
StreptoGramins	Quinupristin/ Dalfopristin					0.5^17^	2-log SCV, other phenotype better^17^		THP-1^17^	
Macrolides	Azithromycin	2.5–6.3^40^		37.8^74^ 45–50^75^	512^74,75^	0.5^74,75^	<1-log^74,75^		THP-1^74^ J774^75^	
	Telithromycin	1.5–2.6^40^		27.9^74^ 8.6–8.7^75^	4^74,75^	0.06^74,75^	<1-log^74,75^		THP-1^74^ J774^75^	
	Erythromycin	0.18–0.28^40^					Only effective immediately, not after 12 h^54^	Normal Mouse Osteoblasts^54^		
	Spiramycin	0.047^40^	1.7–5.3 μg·g^−1^^76^							
Oxazolidinones	Linezolid	0.37–0.51^107^ 0.23–0.51^40^ 0.5^53^ 0.47–0.60^94^	4–9 μg·g^−1^ ^107^ 6.4 μg·mL^−1^ ^17^ 8 μg·mL^−1^^ 53^ 6.3–9.1 μg·mL^−1^^ 94^	0.5^74^	2–4^43^ 4^74^	2–4^43^ 2 SCV & 4 WT^17^ 1^31,53^ 1–16^95^ 2^74^	3-log^53^ <1-log^31,95,97,98^ 1-log immediately and after 7 d^55^ 1-log^74^ SCV 1-log, no difference in phenotype^17^ 2-log^97,99^	MG63^53,98^ pHOBs^55^ MC3T3^99^ NHOst^95^	THP-1^17,74,95^ Primary rat fibroblasts^95^ HUVEC^95^ Calu-3^95^ Primary human keratinocytes^95^ RAW264.7^97^	Sprague-Dawley rats^31^ Kunming mice^97^
	Tedizolid						<1-log^98^	MG63^98^		
	Radezolid					0.25–2^95^	1-log^95^	NHOst^95^	THP-1^95^ Primary rat fibroblasts^95^ HUVEC^95^ Calu-3^95^ Primary human keratinocytes^95^	
Tetracyclines	Doxycycline	0.02–0.85^107^	0.13–2.6 μg·g^−1^ ^107^ 3 μg·mL^−1^ ^41^		0.062 5–0.125^43^	0.125^43^				
Glycolcycline	Tigecycline	0.35–1.8^40^ 0.35^53^	0.3 μg·mL^−1^^ 53^		1^43^	0.125 all phenotypes^17^ 0.062 5–0.25 0.125^53^	4-log^53^ <1-log^108^ SCV 1-log, no difference in phenotype^17^	MG63^53^ pHOBs^108^	THP-1^17^	
Fusidic Acid		0.44–0.93^107^ 0.12–0.94^40^	15–45 μg·g^−1^ ^107^			0.03–0.125 SCV < WT^17^	<1-log SCV much better against WT/revertant^17^		THP-1^17^	
Aminoglycosides	Gentamicin		Detectable^41^	4.4^74^ 6.3–6.8^75^	32-64 SCV & 1 WT^125^ 16^74,75^	0.125 SCV & 0.5 WT^17^ 1 SCV & 0.25 WT^125^ 0.5^74,75^	1-log SCV much better against WT/revertant^17^ <1-log^108,129,141^ 2-log^125^ 1-3-log^126^ 1-log^74,75^ immediately and after 7 d^55^	pHOBs^55,108^ Saos-2^126^ MC3T3^129,141^ BMOC^141^	THP-1^17,74,125^ J774^75^ RAW164.7^129^	C57BL/6 mice^55^ BALB/c mice^129^

### Enzyme inhibitors

#### Fluoroquinolones: ofloxacin, moxifloxacin, levofloxacin, ciprofloxacin, garenoxacin

Fluoroquinolones are a large group of fully synthetic antibiotics that can be divided into four generations. The first generation, e.g., rosaxacin, was effective only against gram-negative bacteria; however, later generations, e.g., moxifloxacin, have a broader antimicrobial spectrum, including gram-positive bacteria.^130^ Fluoroquinolones inhibit prokaryotic topoisomerases II and IV, which inhibits DNA unwinding, leading to strand breakage and a bactericidal effect.^131^

Fluoroquinolones are frequently used for infections, including osteomyelitis, where other antibiotics have failed, due to their broad antimicrobial spectrum and the low incidence of resistance mechanisms, which include a modification of the target enzyme and access to the target.^45,130,132^ However, older fluoroquinolones, especially when used as monotherapies, showed increased levels of resistance.^133^ Therefore, when possible, these should preferably be used in combination therapy.^134^

The penetration into the bone of this group of compounds differed depending on the drug but was possibly sufficient, with bone concentrations between 3–14 μg·mL^−1^ ^41,53^ and MICs between 0.06–0.25 μg·mL^−1^ in the included studies at pH 7 and between 0.125–2 μg·mL^−1^ at pH 5.^17,43,55,74,75,125,135^ This indicates a strong pH dependency (4–8-fold in the in vitro studies) of the efficiency of the drugs, which might impair the treatment of intracellular bacteria, even if the cellular penetration ratios are quite high (between 3.2 and 13.4).^74,75^

In in vitro studies of monocytes and osteoblasts intracellularly infected with *S. aureus*, fluoroquinolones were extracellularly much more effective, but intracellularly were still one of the most effective groups of drugs, with a maximal 2-log reduction of intracellular bacteria compared to 4-log extracellularly for most host cell types.^17,43,53,55,74,75,125,135^ SCVs were 2-log less effectively reduced in number than the WT strain,^125^ while the effect on SCV development induction was inconclusive, with one study observing an induction with moxifloxacin^55^ and another reporting no effect with levofloxacin.^135^ A remarkable characteristic of fluoroquinolones is that they retain effectiveness if the initiation of treatment is delayed^55^ and that a rapid reduction in intracellular bacteria number is observed.^17^ Most studies reported a non- or only slightly dose-dependent effect.^74,75^ The overall effectiveness of the drugs varied among different antibiotics, with moxifloxacin being very effective (3-log reduction)^17,55,125^ and ciprofloxacin showing little effect (1-log reduction).^74,75^

The use of a porous gelatin-hydroxyapatite (HAP) scaffold was found to increase the intracellular concentration of ciprofloxacin and improve the survival of osteogenic cells.^136^ Levofloxacin (2.5%) in acrylic bone cement with added lactose could reduce intraosteoblastic bacteria numbers up to 1.5-log against methicillin-sensitive *S. aureus* (MSSA) and 0.5-log against a MRSA strain; however, no direct comparison to free levofloxacin was performed.^137^ Other studies show a similar reduction of 1-2-log with free levofloxacin.^43,74,135^ The pH-dependent effectiveness of levofloxacin can be manipulated by combining it with hydroxychloroquine to increase the intracellular pH, resulting in a 6.5-fold higher intracellular effectiveness of levofloxacin in osteoblasts.^43^

Overall, the intracellular effectiveness of fluoroquinolones against intracellular *S. aureus* is relatively well studied in vitro. The antibiotic concentrations reached inside osteoblastic cells seemed to be higher than the MICs for *S. aureus*, leading to an overall effective intracellular reduction. Therefore, fluoroquinolones, especially moxifloxacin, should be considered for the intracellular treatment of *S. aureus* in osteomyelitis.

#### Ansamycins: rifampicin, rifapentine, rifabutin

Ansamycins, with rifampicin (or rifampin) by far the most famous example, are commonly used for the treatment of tuberculosis. These are antibiotics that are produced by *Amycolatopsis rifamycinica* and semisynthetically modified.^138,139^

Due to their ability to penetrate well into inaccessible infection loci in the body (e.g., granulomas in tuberculosis, intracellular bacteria) and their high effectiveness against many bacteria (gram-positive, gram-negative and atypical bacteria and mycobacteria), they are used more often in difficult-to-treat infections, such as osteomyelitis, after the failure of other antibiotics.^45,107^ The mechanism of action of ansamycins is the inhibition of DNA-dependent RNA polymerase, inhibiting transcription and therefore the ability to produce proteins as well as reproduce, leading to a bactericidal effect. One main challenge with the clinical use of ansamycins is the rapid acquisition of resistance by the alteration of the binding site of the RNA polymerase, which is found on the *rpoB* gene. To prevent this, ansamycins are frequently combined with other antibiotics.^140^

Rifampicin penetrates the bone well, with penetration rates reported in the range of 0.08 to >1,^40,53,107^ leading to a concentration of approximately 1.3 μg·mL^−1^ in the cortical bone and 6.5 μg·mL^−1^ in cancellous bone.^41^ Furthermore, rifampicin also penetrates well into cells with an intra- to extracellular ratio of 17.6 + /−0.9^74^ and has a lower MIC at lower pH, with an increase up to 8-fold at pH 7,^74,125,135^ indicating that rifampicin could be very effective intracellularly in bone cells. Additionally, both the SCV and WT forms seem to have similar MICs.^125^

For intracellular studies in osteoblasts and monocytes, concentrations between 2–18 μg·mL^−1^ have been used,^17,53–55,125,126,135,141,142^ which are often higher than can be expected in the bone. However, since MICs of ansamycins are often 10-1 000-fold lower than the expected concentration in the bone, this might not impair the results. Studies with ansamycins against intracellular *S. aureus* have contradictory findings. In some studies, the effect against intracellular bacteria was found to be significant, albeit reduced compared to that for extracellular infections,^17,74,125,141^ as evidenced by a 3-log reduction in intracellular SCVs after 72 h compared to that after 24 h against extracellular bacteria^17^ and a 5-log reduction against WT strains, even at low concentrations.^53^ However, other studies reported little or no effect against intracellular bacteria.^74,108,135,142^ The effectiveness against SCVs seemed to be high and similar to that against the WT strain in one study^125^ but was reduced in another study.^17^ Likewise, some studies observed an induction of SCV formation,^135,142^ while others found a reduction or no significant change in phenotype.^53,55^ This could possibly be explained by the high variation among SCVs and the possibly multiple mechanisms, by which conversion to an SCV phenotype occurs. The effect of a delayed start of treatment is also inconclusive. One study showed a significant reduction in effectiveness after a 12 h delayed treatment start post-infection,^54^ while two studies showed no difference in the effectiveness after an immediate treatment start vs. a 7-day delay.^55,142^ Overall, the intracellular effectiveness of ansamycins seems to be similar in osteoblasts^53–55,108,135,143,144^ and macrophages.^17,74,125^

To enhance drug delivery, a study incorporated ansamycins into polymethylmethacrylate (PMMA), which proved to be very effective against biofilms. However, the treatment was effective only against intraosteoblastic bacteria at a dose of 64 μg·mL^−1^, which was cytotoxic to the host cells.^145^ Rifampicin-loaded nanoparticles could reduce intraosteoblastic bacterial numbers more effectively than free rifampicin at multiple dosages. The highest benefit was reached at 10 ng·mL^−1^, with an 18.5-fold better reduction, and at a concentration of 5 ng·mL^−1^, the nanoparticle-loaded treatment was still approximately 5-fold more effective than the free drug.^143^

Preclinical data in rats showed that all ansamycins were very effective against a bacterially inoculated Kirschner wire, with a 4-log reduction in CFU in the bone, without leading to resistance.^146^ Another study in mice showed that rifampicin reduced the number of CFU by approximately 2-log, which was superior to either cefuroxime or gentamicin when given in an acute infection but not at a chronic stage.^55^ A limiting factor of these studies is that effects on the numbers of intracellular CFU were not measured.

Although some study results showed contradictory effects, overall, ansamycins are one of the best studied antibiotic groups for intracellular infections and seem to have a remarkable ability to reduce the intracellular numbers of *S. aureus*. When administered in combination with other antibiotics to prevent the development of resistance, they seem to be a good treatment option for intracellular *S. aureus* infections in osteomyelitis. However, it must be considered that companion drugs need to have a similar penetration and distribution to prevent the development of resistance, which can be challenging in the presence of biofilms or for intracellular pathogens.

A summary of the data relating to antibiotics with bacterial enzyme inhibition as a mode of action is shown in Table 2.

**Table 2:** Overview of enzyme inhibitors tested against intracellular *S. aureus*

Antibiotic Class	Antibiotic	Bone: plasma ratio	Bone concentration	Intra: extracellular ratio	MIC at ca pH5 (μg·mL^−1^ or mg·L^−1^)	MIC at ca pH7 (μg·mL^−1^ or mg·L^−1^)	Intracellular effectivity against *S. aureus*	Model tested		
								bone cell	Other cells	In vivo
Fluoroquinolones	Ofloxacin	0.01–1^40^ 0.5^53^	3 μg·mL^−1^ ^53^		2^43^	0.5^53^ 0.25–.0.5^43^	5-log^53^	MG63^53^		
	Moxifloxacin	0.27–0.49^107^ 0.33–1.05^40^	1.3–1.9 μg·g^−1^^ 107^ 2.8 μg·mL^−1^ ^41^	11.4–13.4^75^ 7.6^74^	0.125–0.5 SCV & 0.125–0.25 WT^125^ 0.25^74,75^	0.125 SCV & 0.25 WT^17^ 0.125 SCV & 0.03–0.06 WT^125^ 0.06^74,75^	3-log^125^ 1.5-log^75^ 1-log immediately and after 7 d^55^ 2-log^74^ SCV 2.5-log^17^	pHOBs^55^	THP-1^17,74,125^ J774^75^	
	Levofloxacin	0.38–0.99^107^ 0.36–1^40^	3–7.4 μg·g^−1^^ 107^ 4.6–10 μg·mL^−1^ ^41^	7^74^	0.5–2^43^ 0.38–0.75^135^ 1^74^	0.125–0.25^43^ 0.13–0.25^135^ 0.25^74^	1-log^43^ 2-log^74,135^	MG63^43,135^	THP-1^74^	
	Ciprofloxacin	0.27–0.66^107^ 0.16–0.42^40^	0.1–2 μg·g^−1^ ^107^ 13.8 μg·mL^−1^^ 41^	5.1^74^ 3.2^75^	1^74^ 4^75^	0.125^74^ 0.125^75^	1-log^74^ <1-log^75^		THP-1^74^ J774^75^	
	Garenoxacin	0.4^40^		9.1^74^	0.125^74^	<0.03^74^	2-log^74^		THP-1^74^	
Ansamycines	Rifampicin	>1^107^ 0.08–0.57^40^ 0.27^53^	5 μg·g^−1^^ 107^ 1.3–6.5 μg·mL^−1^^ 41^ 6 μg·mL^−1^ ^53^	17.6^74^	0.002 SCV & WT^125^ 0.002–0.006^135^ 0.002^74^ 0.000 9–0.007^43^	0.000 5^17^ 0.004^53^ 0.003–0.016 SCV & 0.016 WT^125^ 0.008–0.016^135^ 0.008–0.031^142^ 0.007 5^74^ 0.007 5–0.03^43^	3-log SCV, more effective in other phenotypes^17^ 5-log^53^ 1-log^74,125^^,^^135,145,146^ Cleared bacteria immediately,^126^ not 12 h delayed^54^ <1-log^108,141,142^ >1-log, best in study, more effective after 7 d^55^ 2-log at 5 ng/ml, up to 9-log at 50 ng/l^143^ 3-log^144^	MG63^53,135,142,144^ NHOst^54^ Normal mouse OBs^54^ pHOBs^55,108,145^ Saos-2^126^ MC3T3^141,143^	THP-1^17,74,125^	C57BL/6 mice^55^ Wistarrats^146^
	Rifapentin				<0.003 5–0.007^43^	0.007–0.015^43^ 0.062–0.031^142^	<1-log^142^	MG63^142^		Wistar rats^146^
	Rifabutin				0.125–0.5^43^	<0.003 5–0.007^43^ 0.031–0.062^142^	1-log^142^ 4-log^146^	MG63^142^		Wistar rats^146^

### Cell wall disruptors

#### Beta-lactam inhibitors: oxacillin, ceftaroline, cefuroxime, flucloxacillin, ampicillin, dicloxacillin, nafcillin, penicillin V, ertapenem, meropenem

Beta-lactam inhibitors are one of the longest in-use modern antibiotics since the discovery of penicillin and have now evolved to be one of the most complex antibiotic groups, with multiple subgroups including penicillins, cephalosporins, monobactams, carbapenems and carbacephems, of which some have further subgroups.^147,148^ Beta-lactam antibiotics target the cell wall, the principal cell integrity structure in gram-positive bacteria, with the main mechanism of action being irreversible binding to penicillin-binding proteins (PBPs) during peptidoglycan synthesis, leading to inhibition of cell wall synthesis.^147^ This does not cause immediate bacterial cell death but hampers replication, as well as cell wall repair, and the effect is therefore classified as secondary or indirect bactericide. Bacteria can become resistant to some beta-lactam antibiotics due to the induction of a beta lactamase or methicillin resistance gene (*mecA*).^149^

Most beta-lactam antibiotics reach only very low concentrations in the bone, with approximately 4 μg·mL^−1^ for oxacillin, ceftaroline, cephalexin and dicloxacillin. Exceptions to this include flucloxacillin, reaching 89.5 μg·mL^−1^, and ertapenem, reaching 27–83 μg·mL^−1^.^41,107,150^ The bone:plasma ratio can be between 0.04–0.71 but is approximately 0.2 for most beta-lactam antibiotics.^40,53,107^ The intracellular absorption is very low, with an intra- to extracellular ratio of 1.0–4.0.^74^ Concentrations used in most studies are oriented on the maximal plasma concentrations, which are much higher than concentrations in the bone, e.g., 63–64 μg·mL^−1^ of oxacillin, 47.6 μg·mL^−1^ ampicillin and 100 μg·mL^−1^ cefazolin.^17,74,141^

The very low bone accumulation and even lower mammalian cell penetration seem to make them unsuitable treatment options for bone infections; however, some beta-lactam antibiotics are regularly used in jaw surgery, as well as in the treatment of osteomyelitis.^45,107,151^

Multiple beta-lactam antibiotics showed a much lower effectiveness, up to a 3-log difference, against *S. aureus* intracellularly than extracellularly,^17,74,141^ with generally very low effectiveness, often less than or approximately 1-log reduction.^58,74,141,150,152,153^ The only reported exception is oxacillin, which could reduce the number of intracellular bacteria by up to 4-log but with a reduced effectiveness against SCVs.^17,53,74^ Due to the wide variation in experimental design, including the host-cell type used, for each antibiotic, no conclusion about the influence of the cell type can be drawn here.

Beta-lactam antibiotics permeate the cell membrane well due to their small size; however, they also leave the cell easily and do not accumulate due to their acidic character.^42,154^ This has been addressed by combining beta-lactam antibiotics with efflux inhibitors to increase the intracellular effectiveness by 1 to 3-log.^152^

The effect of beta-lactam antibiotics on the emergence of SCVs is inconclusive: one study showed a reduction with oxacillin but induction with ceftaroline,^53^ and another showed no induction with either cefuroxime or flucloxacillin.^55^ Beta-lactam antibiotics displayed dose- and time-dependent efficiency,^53,74^^,^ with immediate treatment being more effective than delayed treatment.^55^

Interestingly, beta-lactam antibiotics were more active against MRSA intracellularly than extracellularly due to acetylation of the resistance protein PBP 2 at the intracellular pH 5, which leads to a loss of function.^155^ This was apparent in the observed reduced MIC at pH 5 compared to that at pH 7.^43,74,150,153^ Additionally, oxacillin reduced the level of an intracellular MRSA strain by 75%, while only a 45% reduction was achieved with the corresponding MSSA strain; this effect was not observed with cefazolin, which does not show a differential efficiency.^153^

Nafcillin contained in PLGA nanoparticles could clear an intracellular infection of osteoblasts.^156^ The nanoparticles alone reduced the CFU count by 0.5-log, and the additional antibiotic treatment was enough to clear the infections in the two formulations. A limitation of this study was the lack of a control treatment of free nafcillin. In another study, the inhibitory effect of nafcillin was approximately 1-log in macrophages.^74^ Penicillin G phospholipid nanoparticles could increase the intracellular uptake up to 5-fold (up to 10% in total) in an epithelial cell line model, leading to an increased intracellular antibiotic activity up to 7-fold, which in total still resulted in a less than 1-log CFU reduction.^157^

In an in vivo infection study, cefuroxime did not significantly reduce the CFU count in an acute or chronic state.^55^

Thus, while beta-lactam antibiotics are an important group of antibiotics with many indications, the currently available data suggest that they are not an effective choice for the treatment of intracellular *S. aureus* infections in osteomyelitis, with the exception of oxacillin, as discussed above.

#### Glycopeptides: vancomycin, oritavancin, teicoplanin, telavancin

Vancomycin, which is produced by the bacterium *Amycolatopsis orientalis,*^158^ was considered a ‘game changer’ in the antibiotic treatment of gram-positive bacteria and was for a time one of the most successful treatments against antibiotic-resistant strains, especially MRSA. However, as with any new antibiotic, some bacteria developed and transferred mechanisms of resistance, leading to the development of new generations of semisynthetic glycopeptides.^159^

Like beta-lactam antibiotics, glycopeptides are bacterial cell wall inhibitors. While beta-lactam antibiotics bind to PBP receptors, glycopeptides bind to the D-Ala-D-Ala moiety of the substrate, preventing enzyme binding and leading to insufficient cell wall synthesis.^160^

To become resistant to glycopeptides, bacteria can vary the D-Ala-D-Ala moiety to reduce the antibiotic affinities up to 100-fold. This can be an intrinsic or acquired mechanism, most characterized in VRE but also in *S. aureus* and vancomycin-resistant *S. aureus* (VRSA). Vancomycin-intermediate *S. aureus* (VISA) has an increasing cell wall thickness, trapping vancomycin molecules, which can be overcome by increasing the dose of vancomycin.^160,161^

Glycopeptide antibiotics penetrate the bone well, reaching concentrations of 3–65 μg·mL^−1^, which are much higher than the MICs for most *S. aureus* strains (0.015–2 μg·mL^−1^),^17,31,43,53,74,75,125^ with bone:plasma ratios between 0.05–0.97. They also accumulate well but slowly intracellularly, with intra- to extracellular concentration ratios between 6.3–344.^74^ The MICs are not or only slightly pH dependent;^43,74,75^ however, the antibacterial effect is strongly time dependent, often only showing after a delay of 24 h,^17,74^ and the MBCs are much higher than the corresponding MICs (up to 16-fold),^74^ which might be a catalyst for inducing persister phenotype development. Even though the agents of this group share some main characteristics, there are also several differences, especially between vancomycin and the newer agents. In the studies reviewed, vancomycin was used at higher concentrations than can be expected in the bone but still showed a maximal inhibition effect of a 2-log reduction, independent of the cell type used.^17,74,125,141,144,162,163^ It was possible to increase the effect in combination with other agents, such as ansamycins,^146^ bacteriophages,^144^ peptide-conjugated antimicrobial peptide TAT-KR12^164^ or efflux pump inhibitors,^152^ but not with liposomes or pegylated liposomes.^163^ Interestingly, vancomycin showed increased antimicrobial potency when used after 7 days compared to that at an immediate treatment time.^55^

The effectiveness of glycopeptides against SCVs is inconclusive. Some studies showed a 1-log reduction against SCVs,^17^ while another showed no effectiveness.^125^ The MICs against SCV and WT forms seemed to be similar,^17,125^ and an induction of SCV formation could be found in some cases^53^ but not in others.^55^

In contrast to vancomycin, newer glycopeptides, in particular oritavancin and teicoplanin, showed a very high intracellular effectiveness against *S. aureus*, with a 3- to 4-log reduction at bone-relevant concentrations,^17,53,74,75,125^ often being the most effective drug in each of the respective studies. Most studies were conducted in macrophages; hence, a cell type-dependent effect could not be evaluated. However, teicoplanin used in both macrophages and osteoblasts was found to be 2- to 3-log more effective in osteoblasts.^53,74^ The MICs against SCVs were lower, between 0.03–1 μg·mL^−1^ for oritavancin and 0.125 μg·mL^−1^ for teicoplanin, compared to vancomycin with MICs of 0.25–2.^74,125^ In infected cells, oritavancin reduced the number of SCVs by 3-log, which was comparable to the reduction of the number of WT,^17^ and showed no induction of SCV development.^53^ Telavancin, however, is more comparable with vancomycin in its effectiveness than with the other newer agents.^17^

Three enhanced drug delivery systems could improve the intracellular effectiveness of vancomycin. In an osteoblast model, vancomycin-loaded N-trimethyl chitosan nanoparticles (TMC NPs) and vancomycin-loaded N-trimethyl chitosan nanoparticle poly-trimethylene carbonate (TMC-NP-PTMC) showed an improved antibacterial effect of up to 1-log, with TMC-NP-PTMC being more effective. It should be noted that the nanoparticles were loaded with 50 μg·mL^−1^ vancomycin, which is approximately 10-fold higher than the concentrations of vancomycin usually found in the bone.^165^ Another vancomycin drug delivery system using mannosylated exosomes (MExoVs) increased intracellular CFU reduction by 0.5-log in macrophages and approximately 1-log in epithelial cells.^166^

In vivo, the treatment of intracellular *S. aureus* in infected rats with osteomyelitis was successful with vancomycin in 15%–20% of animals.^31^ This success rate could be increased by using drug delivery systems, such as TMC-NP-PTMC, which showed a < 1-log improvement,^165^ and MExoV increased the effectiveness of vancomycin by 2-log in mouse kidneys.^166^

Even with the emergence of vancomycin resistance, glycopeptides still seem to be a very effective antibiotic group against *S. aureus*, including for intracellular infections, specifically with newer agents, such as oritavancin. The preclinical data for vancomycin also show that drug delivery systems could increase the effectiveness further. Additional studies are required in bone cell models to inform a recommendation for the treatment of intracellular *S. aureus* in osteomyelitis.

#### Fosfomycin

Fosfomycin is a cell wall inhibitor, first isolated by screening broth cultures of *Streptomyces fradiae*, and is a competitive antagonist of phosphoenolpyruvate, which is required for the synthesis of n-acetyl-muramic acid, a component of peptidoglycan.^167^ It is effective against gram-positive and gram-negative bacteria, including MDR bacteria such as MRSA and VRE and multidrug-resistant gram-negative bacteria; however, resistance against fosfomycin is also common. Mechanisms of resistance can be inherited, such as altered enzyme structure or enzymatic pathways, or acquired, in the case of cell transporter modifications, mutations, and enzymic inactivation.^168^

The main indication for fosfomycin is as a single-dose treatment of urinary tract infections, especially those caused by *E. coli*. Fosfomycin has also been used in osteomyelitis, albeit uncommonly.^107,169,170^

Fosfomycin has a bone:plasma ratio of 0.13–0.45, which leads to bone concentrations of 4 μg·mL^−1^ or 16–96 μg·g^−1^.^40,53,107^ It seems to be very effective (up to a 3-log reduction intracellularly, independent from the dose) when used immediately after the infection of bone cells but loses its effectiveness when given after 7 days. The influence on SCV emergence is inconclusive.^53,55^

Since there are only two studies that have reported the efficacy of fosfomycin against intracellular *S. aureus,*^53,55^ further studies are required to establish its utility as a treatment option for osteomyelitis.

#### Lipopeptides: daptomycin

Daptomycin is produced by fermentation by the actinobacterium *Streptomyces filamentosus.*^171^

It is clinically used when other antibiotics fail due to its effectiveness against MDR gram-positive bacteria, including *S. aureus, S. pyogenes* and enterococci, including VRE, as well as its effectiveness against biofilms.^172,173^

The molecule is a cyclic lipopeptide with a hydrophilic ring and lipophilic end, which enables daptomycin to penetrate the gram-positive cell wall and cause membrane depolarization.^173^

Resistance to daptomycin is rare but emerging with its increased use. Mechanisms of resistance thus far characterized are cell wall adaptations that lead to reduced binding of daptomycin by either diversion from the binding site or electrostatic repulsion.^174,175^

Daptomycin is used off-label for osteomyelitis treatment, and in clinical trials, cure rates range between 63%–89%.^45,107,172,176,177^

The bone:plasma ratio of daptomycin is between 0.07–0.24, leading to a bone concentration of 5 μg·mL^−1^ or 5 μg·g^−1^, which is similar to the plasma concentration of 4–11 μg·mL^−1^.^53,107,153^ This is higher than the MICs observed in selected in vitro studies of 0.064–4 μg·mL^−1^, which seem to be slightly increased by a lower pH.^17,43,53,125,153^ Even though the MICs varied considerably, no dependence on pH, phenotype or methicillin sensitivity could be seen.

In most studies, daptomycin could not clear intracellular *S. aureus* infections effectively, up to a maximum of 2-log and only after long treatments with very high concentrations up to 100 μg·mL^−1^, while it was very effective against extracellular bacteria with up to a 6-log reduction in CFU.^17,43,53,125,153,178^ In one study, a significant intracellular reduction in the number of *S. aureus* was observed when daptomycin was given immediately but not after a 7-day delay.^55^ A limitation, however, was that a concentration of 60 μg·mL^−1^ was used, which is much higher than can be expected inside the bone, and even though the reduction was significant, it was less than 2-log. The effect of daptomycin on SCVs is unclear, since one study found a reduction in SCV emergence,^53^ while another observed an induction, even though this was not significant.^55^ The effectiveness against SCVs seemed to be reduced compared to that against the WT and reverted forms.^17^ One factor for the effectiveness of daptomycin in the bone might be the high local concentration of calcium, which has been taken into consideration in only one study.^125^ The limited number of studies available indicates a higher potency for the treatment of intracellular infections in macrophages than in bone cells.

There have been some efforts to improve the drug delivery of daptomycin to bone infections. One study showed that encapsulation in poly-(methyl-methacrylate)-eudragit microparticles achieved a higher daptomycin concentration over five days in vitro in osteoblasts and in vivo in mice, resulting in improved bacterial clearance and healing than with free daptomycin.^178^ An improvement in effectiveness of 2.7-fold was reached by combining daptomycin with hydroxychloroquine to increase the intracellular pH.^43^

Despite the high effectiveness of daptomycin against MDR bacteria and the relatively high bone concentration achievable, it appears to be less effective against intracellular *S. aureus* and therefore might not be the best treatment option for a persistent *S. aureus* infection in osteomyelitis.

A summary of the data relating to antibiotics with bacterial cell wall disruption as a mode of action is shown in Table 3.

**Table 3:** Overview of cell wall disruptors tested against intracellular *S. aureus*

Antibiotic Class	Antibiotic	Bone: plasma ratio	Bone concentration	Intra- to extracellular ratio	MIC at ca pH5 (μg·mL^−1^ or mg·L^−1^)	MIC at ca pH7 (μg·mL^−1^ or mg·L^−1^)	Intracellular effectivity against *S. aureus*	Model tested		
								bone cell	Other cells	In vivo
**Beta-lactam antibiotics**	**Oxacillin**	0.1^107^ 0.11^40^ 0.17^53^	4 μg·mL^−1^ ^41^ 10 μg·mL^−1^ ^53^	4.0^74^	<0.015–0.062 5^43^ 0.06^74^ 0.016 MSSA, 0.023 MRSA^153^	0.125 SCV & 0.5 WT^17^ 0.094^53^ <0.015–0.062 5^43^ 0.125^74^ 0.25 MSSA, > 256 MRSA^153^	SCV 1-log, more effective against the WT^17^ 4-log (dose depending), SCV emergence reduction^53^ 2-log, time depending^74^ <1-log^153^	MG63^53,153^	THP-1^17,74^	
	**Cefatroline**	0.19^53^	4 μg·mL^−1^^ 53^		0.006 MSSA, 0.008 MRSA^153^	0.19^53^ 0.125 MSSA, 0.25 MRSA^153^	2-log, SCV induction^53^ <1-log, similar MRSA/MSSA^153^	MG63^53,153^		
	**Cefuroxime**	0.04–0.55^40^					<1-log immediately, no effect after 7d^55^	pHOBs^55^		C57BL/6 mice^55^
	**Cefazolin**	0.75–0.37^107^ 0.18^40^	3–30 μg·g^−1^ ^107^ 4.2 μg·mL^−1^^ 41^		0.03–0.125^43^	0.062 50–0.5^43^	No effect^58,141^	UMR106^58^ MC3T3^141^		
	**Flucloxacillin**		89.5 μg·mL^−1^^ 41^				<1-log immediately, no effect after 7d^55^	pHOBs^55^		
	**Ampicillin**	0.17–0.33^107^ 0.11–0.71^40^	12–20 μg·g^−1^ ^107^	1.0^74^	0.03^150^ 0.03^74^	0.06^74^ 0.07^150^	1-log^74^ No effect (1 h)^141^ 1-log, time depending less dose depending^150^	MC3T3^141^	THP-1^74,150^	
	**Dicloxacillin**		3.8 μg·mL^−1^ ^41^				ns (ca log1) decrease of bacteria, but can be increased by adding efflux inhibitors (piperine or cyanide m-chlorophenyl hydrazone)^152^	Saos-2^152^		
	**Nafcillin**			2.6^74^	0.06^74^	0.25^74^	1-log^74^		THP-1^74^	
	**Penicillin V**			1.2^74^	<0.015^74^	0.06^74^	1-log^74^		THP-1^74^	
	**Penicillin G**						<0.1-log^157^		A594^157^	
	**Ertapenem**	0.13–0.19^40^			0.06^150^	0.11^150^	1-log, time depending less dose depending^150^		THP-1^150^	
	**Meropenem**				0.06^150^	0.15^150^	1-log, time depending less dose depending^150^		THP-1^150^	
**Glycopeptides**	**Vancomycin**	0.05–0.21^107^ 0.05–0.97^40^ 0.21^53^	1.1–3.6 μg·mL^−1^^ 107^ 3.8–4.5 μg·mL^−1^ ^41^ 6 μg·mL^−1^ ^53^	6.3^74^	1 SCV & 0.5 WT^125^ 0.125–1^43^ 1^74^	0.5 SCV & WT^17^ 1.5^53^ 1 SCV & 0.5 WT^125^ 0.25–2^43^ 1^74^ 2^31^	SCV, in beginning growth, max 1-log, WT much more effective^17^ Ns effect^141,162,164^ Max 1-log^31,125,144,152,166^ immediately & after 7d^55^ 1-log dose and time dependent^74^ Ns bacterial growth^53^ 2-log^163^	MG63^53,144^ pHOBs^55^ MC3T3^141^ Saos-2^152^	THP-1^17,74,125,163^ A594^166^ J774^162^ RAW264.7^164,166^	Sprague-Dawley rats^31^ Kunming mice^166^
	**Oritavancin**		27–65 μg·mL^−1^ ^41^	148^74^ 336–344^75^ (highest by far)	0.25^74,75^ 0.125 SCV & 0.25–1 WT^125^	0.015 SCV & 0.03 WT^17^ 0.07^74^ 0.25^75^ 0.03–0.06^125^	3-log against SCV (best drug of 13)^17^ 3-log strongly time & dose dependent^74^ 4-log dose depending^125^ 3.5-log all phenotypes^75^		THP-1^17,74,125^ J774^75^	
	**Teicoplanin**	0.5–0.85^40^ 0.21^53^	3 μg·mL^−1^ ^53^	7.4^74^	0.0625–2^43^ 1^74^	0.25–0.5^43^ 1.5^53^ 1^74^	3-4-log^53^ <1-log^74^	MG63^53^	THP-1^74^	
	**Telavancin**					0.125 all phenotypes^17^	<1-log SCV, WT better^17^		THP-1^17^	
**Fosfomycin**		0.25^107^ 0.13–0.45^40^ 0.35^53^	16-96 μg·g^−1^^ 107^ 4 μg·mL^−1^ ^53^		1–16^43^	2^53^ 0.25–8^43^	3-log no dose depending^53^ Immediately effective not after 7d^55^	MG63^53^ pHOBs^55^		
**Lipopeptides**	**Daptomycin**	0.07^107^ 0.24^53^	5 μg·g^−1^^ 107^ 5 μg·mL^−1^^ 53^		0.5 SCV & WT^125^ 0.75 MSSA, 0.19 MRSA^153^ 4^43^	0.125 SCV & WT^17^ 0.19^53^ 0.5–2 WT, 0.25–0.5 SCV^125^ 0.064 MSSA, 0.19 MRSA^153^ 1–2^43^	SCV 1-log, WT better^17^ Ns/ bacterial growth^43,53,153^ 2-log^125^ <1-log immediately, after 7d ns^55^ <1-log^178^	MG63^43,53,153,178^ pHOBs^55^	THP-1^17,125^	C57BL/6 mice^178^

## Discussion

**Fig. 2:**
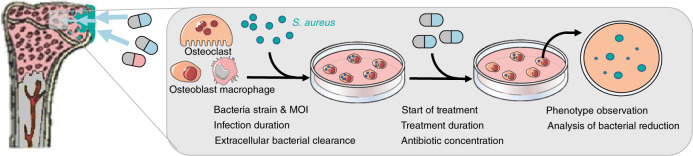
Overview of the methodology of intracellular infection assays

Comparing all studies of intracellular infections included in this review, we noticed a wide variety in methodologies employed, and a lack of a standardized model for testing effectiveness in any infection context, especially osteomyelitis, was clearly apparent, as visualized in Fig. 2. Some differences were minor and might even serve to increase the validity of related studies when examined together, such as the diversity of cell types, number of bacterial species and strains, duration of infection, duration of treatment and concentration of antibiotics used. Other variables seem to impact the quality of the published studies, making them less comparable. For example, it was frequently unclear whether and how extracellular bacteria were removed before treatment. In some studies, the extracellular bacteria were removed before treatment with gentamicin,^54,143,145,152,156,164,166^ daptomycin^179^ or lysostaphin.^53,55,58,98,108,126,135,137,142,153,163,178^ In other studies, an extracellular bactericidal agent was used during the entire treatment period.^43,144^ In additional studies, the cells were only washed^57,99^ or immediately treated^141,165^ without treating the extracellular bacteria separately. On the one hand, the killing or removal of extracellular bacteria ensures that only the effect on intracellular bacteria is observed, while on the other hand, agents used to clear extracellular bacteria may also impact intracellular bacteria.

Even though the variety of dosages of the antibiotics used gives a broader insight into the effectiveness of the treatment, using concentrations multiple times higher than can be expected to be achieved in the bone could render findings less meaningful. The different antibiotic concentrations used can be related to the experimental approach employed, with some studies based on the actual measured MIC in vitro,^54,74,125,142,152,162^ while others chose the concentrations based on the maximum plasma/serum^17,74,75^ or bone^53,135,153^ concentrations observed in vivo. Ideally, both factors need to be considered. Using a concentration of antibiotic that cannot be achieved in the bone does not lead to results relevant for treating osteomyelitis, and using concentrations that are lower than the MIC are unlikely to establish an effective treatment and are more likely to induce adverse effects, for example, phenotypic adaptation, antibiotic resistance, autophagy or an adverse immune response. It is postulated that a concentration 4-fold higher than the MIC should be reached in infected tissue to maximize the chance of an effective treatment and minimize the risk of unintended antibiotic effects.^180^ Consequently, we advocate using antibiotic concentrations in a model that are at least 4-fold the MIC and lower than the maximal bone concentration.

Another main difference between the studies identified is the choice of cell model, with many using macrophages and cell lines, which behave differently from bone cells and primary cells in the context of *S. aureus* infection. Macrophages, as professional phagocytes, can be infiltrated by up to 100-fold greater numbers *of S. aureus* than osteoblasts, and *S. aureus* showed high intracellular survival after up to 8 days post-infection in osteoblasts.^181^ In comparison to other nonprofessional phagocytic cell types, primary osteoblasts were found to internalize fewer bacteria but also exhibit a higher infected cell survival rate 24 h post-infection and a high intracellular survival rate of *S. aureus* 7 days post-infection. Furthermore, there was a great difference in the behavior of an osteoblastic cell line (CRL-11372) compared to that of primary osteoblasts. The latter had much lower cell death rates when infected and more bacteria persisting in phagosomes than the transformed cell line.^29^ This indicates that to determine the effectiveness of antibiotic treatment for osteomyelitis, it is crucial to use an appropriate bone cell model. While primary cells are intuitively more likely to yield more clinically relevant data, cell lines have the benefit of greater availability and reproducibility.

Some studies evaluated a treatment as being significantly effective when a certain percentage, e.g., 50% reduction, in the number of CFU was achieved,^55,57,58,142,153,157,165,178^ while others considered the linear reduction in the CFU count,^126,141,156,163,179^ logarithmic bacterial number reduction,^17,53,74,75,95,97,125,144,146,150,162,166^ or only host-cell survival.^136^ Since complete clearance is rarely achievable and a bacterial reduction of less than 1-log does not seem to be useful in an infection context, in this article, an effort was made to compare results at a logarithmic level. Furthermore, especially in drug delivery studies, there is often no effective control employed, for example, when comparing a novel drug delivery system with the conventional form,^137,156,165^ and in vivo studies rarely take intracellular bacteria into account.

Another main methodological problem is that most studies included used an immediate treatment after infection, with only three of the studies choosing delayed treatments of 12 h^54^ or 7 days.^55,142^ Most treatments are limited to a short period of exposure time (24 h), while chronic osteomyelitis patients are often treated for weeks or months after the infection manifests. Therefore, conclusions for the translatability of experimental infection findings into clinical practice are limited.

Another limiting factor might be the overall survival of the cells, since mostly macrophages and osteoblasts (20 studies) were used, which have a limited lifespan in vivo and an even shorter lifespan in vitro. Only one study used osteoclasts,^141^ and none considered osteocytes. Since osteocytes are by far the longest living and most abundant bone cell type in vivo and are proven to be able to be serve as a reservoir for *S. aureus* in vivo,^34^ ex vivo and in vitro, as well as being able to survive intracellular infection in an in vitro model over longer periods,^25^ they seem to be a highly relevant cell type. Surprisingly, an osteocyte infection model has only recently been reported,^182^ and while this contained some proof-of-concept data concerning antibiotic effectiveness, detailed studies have not yet been reported. Although a rat study demonstrated that most intracellular bacteria in an open wound model are found inside phagocytes,^31^ this does not mean that this cell type is the most relevant in the intracellular persistence of *S. aureus* in osteomyelitis. Cells of the highest relevance are most likely osteocytes, as by virtue of their location in the lacunocanalicular network of hard bone tissue, they provide the most difficult-to-treat reservoir of bacteria and are the longest lived, best interconnected and the most remote from the vasculature in osteomyelitis.

Furthermore, only a few studies have considered the phenotypic adaptation of *S. aureus* to SCVs, which seems to be an important factor in intracellular persistence^24,55,183^ and could be a major reason for antibiotic treatment failure. Only one study evaluated the effectiveness of treatments against SCVs compared to that against the corresponding WT strain,^17^ and only four took the induction of SCV formation by antibiotic treatments into account.^53,55,135,142^

A question that arises from this review is that if there are many contradictory or at least variable results in intracellular in vitro models, can we draw conclusions regarding the intracellular effectiveness of antibiotics in clinical osteomyelitis from extracellular infection models, the bone concentration of an antibiotic and its (host) cellular penetration? To answer this question, we must examine how well these individual factors can predict the intracellular outcome. Barcia-Macay et al. investigated the intracellular accumulation of antibiotics, their intracellular effectiveness and whether the former is a good predictor of the latter. This was found to not be the case. For example, oritavancin accumulated 148% and moxifloxacin 7.6% intracellularly, but they had a similar intracellular activity.^74^ This was also found by Seral et al.,^75^ who additionally examined the relationship between the extracellular and intracellular antibiotic effectiveness, which were found to be independent of each other. This shows that intracellular antibiotic effectiveness depends on factors other than simply the concentration and extracellular activity, which are currently unknown but could include the colocalization of the antibiotic and bacteria, host cell metabolism (e.g., autophagy, antibiotic adsorption to proteins, metabolism of the antibiotic) or intracellular bacterial adaptive mechanisms, such as SCV formation, which are likely to influence antibiotic susceptibility. To include all these unknown factors, the best approach for now appears to be the use of intracellular in vitro models. However, to obtain comparable results, standardized and well-characterized models for the various bone cell types are needed.

**Fig. 3:**
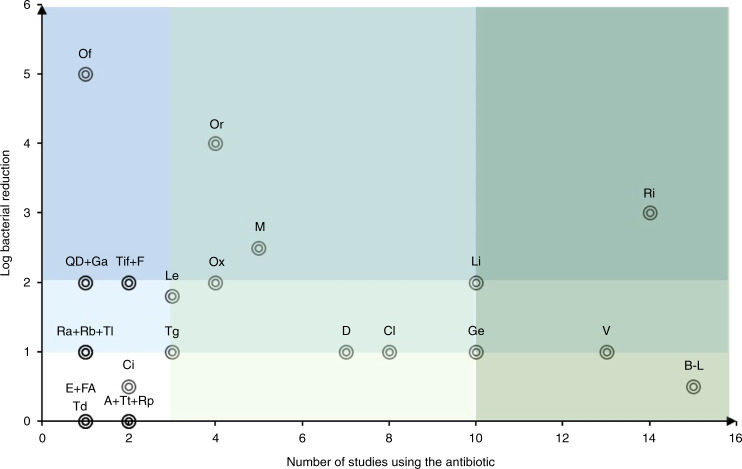
A representation of the effectiveness of antibiotics against intracellular S. aureus and the number of studies that tested the antibiotic. A typical log-reduction value for each antibiotic formulation, which was either reported or gauged from data included in the study in question, was assigned (y-axis) and plotted against the number of studies contributing to these values (x-axis). Supporting data for this analysis are detailed in Tables 1–3. Dark blue zone = high effectiveness, light blue zone = medium effectiveness, white zone = low effectiveness. Dark green zone = highest number of studies, light green zone = low number of studies, white zone = individual studies. A = azithromycin, B-L = beta-lactam antibiotics (except oxacillin), Ci = ciprofloxacin, Cl = clindamycin, D = daptomycin, E = erythromycin, F = fosfomycin, FA = fusidic acid, Ga = garenoxacin, Ge = gentamicin, Le = levofloxacin, Li = linezolid, M = moxifloxacin, Of = ofloxacin, Or = oritavancin, Ox = oxacillin, QD = quinupristin/dalfopristin, Ra = radezolid, Rb = rifabutin, Ri = rifampicin, Rp = rifapentin, Td = tedizolid, Tg = tigecycline, Ti = teicoplanin, Tl = telavancin, Tt = telithromycin, V = vancomycin

From the in vitro data regarding the intracellular effectiveness of antibiotics against *S. aureus* that were considered here and which include, to the best of our knowledge, the extant reports of infected bone cell models, only limited recommendations for the treatment of intracellular *S. aureus* infection in osteomyelitis can be made. The most effective antibiotics thus appear to be rifampicin, oritavancin, linezolid, moxifloxacin and oxacillin. Antibiotics that seemed to be effective but have limited supportive evidence are teicoplanin, quinupristin/dalfopristin, garenoxacin, fosfomycin and ofloxacin. Treatment options that might be useful but for which there is little evidence are tigecycline, levofloxacin and doxycycline. In contrast, antibiotics that are, with some evidence, ineffective are clindamycin, gentamicin, vancomycin and daptomycin. Additionally, albeit with little evidence, nonendorsed treatment options would be macrolides, tedizolid, telavancin, radezolid, fusidic acid, rifapentine, rifabutin and beta-lactams, with the exception of oxacillin. A graphical representation of the effectiveness of individual antibiotics found in the included studies, based on the typical log-reduction in the number of CFU, in relation to the number of supporting studies is depicted in Fig. 3.

Since this systematic review focused on evaluating antibiotics that have been studied for their intracellular effectiveness, we did not discuss antibiotics that might be effective but have yet to be tested in an adequate model. This included older antibiotics, such as doxycycline, which can reach a concentration in the bone of up to 3 μg·mL^−1^, compared to MICs for *S. aureus* of 0.062 5–0.125 μg·mL^−1^. The folate synthesis blocker combination TMP-SMX seems to be another promising candidate since it showed a high intracellular effectiveness against *S. aureus* in neutrophils^44^ and is effectively used in combination treatments in MRSA infections.^184,185^ However, an increase in resistance to TMP-SMX in MRSA has been reported in recent years.^186,187^ Additionally, the possible induction of SCV formation must be taken into consideration. Furthermore, newer antibiotics that did not appear in our search at the time of the study should be evaluated for their potential intracellular effectiveness. For example, omadacycline, a novel tetracycline, seems to be effective against MRSA in osteomyelitis in a mouse model^188^ and intracellularly against *Legionella pneumophila,*^189^ even though its effectiveness seems to be pH dependent.^190^

In addition to the effectiveness of individual drugs, combination treatments must also be taken into consideration. Combination treatments can, on the one hand, reduce the risk of the development of resistance, such as that to rifampicin, if they have similar drug distribution and penetration properties but also have pharmacokinetic effects on each other, such as altered metabolism. On the other hand, combination therapies can also increase the risk of resistance development and/or hamper the activity of each other if not chosen appropriately, for example, in the case of rifampicin used in combination with fusidic acid.^112^

Furthermore, the effectiveness of drugs intracellularly could be improved by smart drug delivery systems such as nanoparticles,^57,99,129,143,156,157,165,166^ exosomes,^97^ porous gelatin-hydroxyapatite scaffolds,^136^ advanced acrylic bone cements,^137^ microparticles,^178^ or a combination to manipulate the pH to a more favorable level for the antibiotic, such as with a hydroxychloroquine combination.^43^

Overall, based on current evidence, we know very little about effective treatments for intracellular *S. aureus* infections in the context of osteomyelitis. This needs to be addressed given the increased incidence of osteomyelitis, best characterized for PJI, with no recent improvement in treatments and associated high relapse rates. One major problem is the lack of standardized models, including in vitro models relevant to human osteomyelitis. Antibiotics that seem to be promising, such as teicoplanin, quinupristin/dalfopristin and ofloxacin, need to be studied in more detail, as should those antibiotics that have shown effectiveness in at least some studies, such as rifampicin, oritavancin, linezolid, moxifloxacin and oxacillin. Furthermore, antibiotics such as doxycycline, omadacycline and TMP-SMX have yet to be tested against *S. aureus* in an intracellular osteomyelitis model. These should be investigated in bone-relevant models and for their effectiveness against SCVs. To increase clinical relevance, they should also be studied in the context of delayed treatment for acute infections and over longer time periods for chronic infections. Finally, combinations of antibiotics in accordance with their clinical application should also be examined in more detail to account for differential intracellular effectiveness and inappropriate drug interactions. More research is urgently required to improve the evidence for potential treatments and, ultimately, the clinical outcome for osteomyelitis patients.
